# Supplementary material for: An Appraisal of Potential for Sowing of Nasturtium officinale into Streams to Mitigate Nutrient Pollution in Eastern Scotland
Source: Int J Environ Res Public Health. 2020 Jan 31;17(3):895. doi: 10.3390/ijerph17030895 (PMC7037887; doi:10.3390/ijerph17030895)
Supplement: Supplementary file 1 [file ijerph-17-00895-s001.pdf]

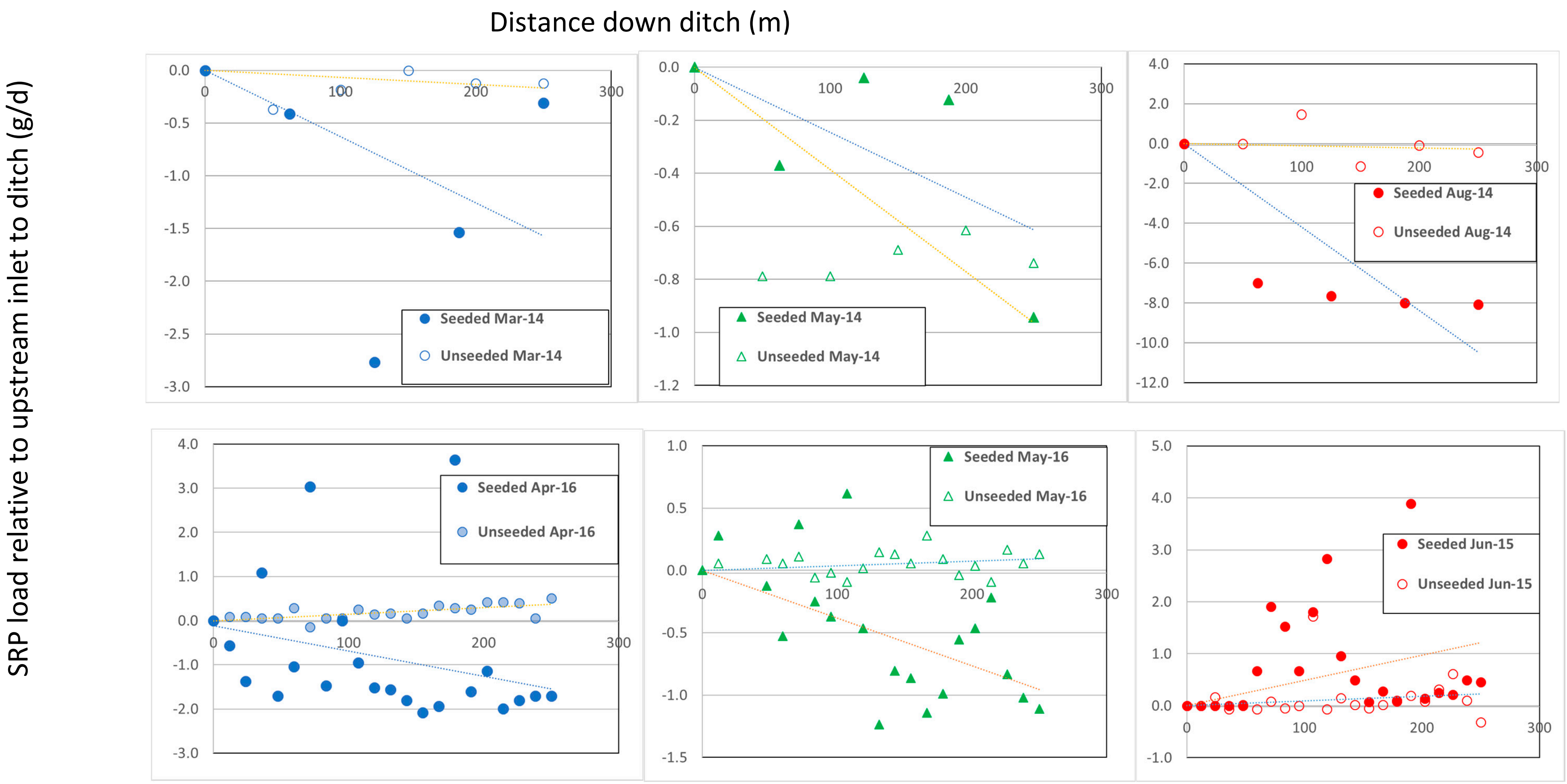

Supplementary data 1a. SRP load change along watercress seeded and unseeded ditches from top end of ditch.

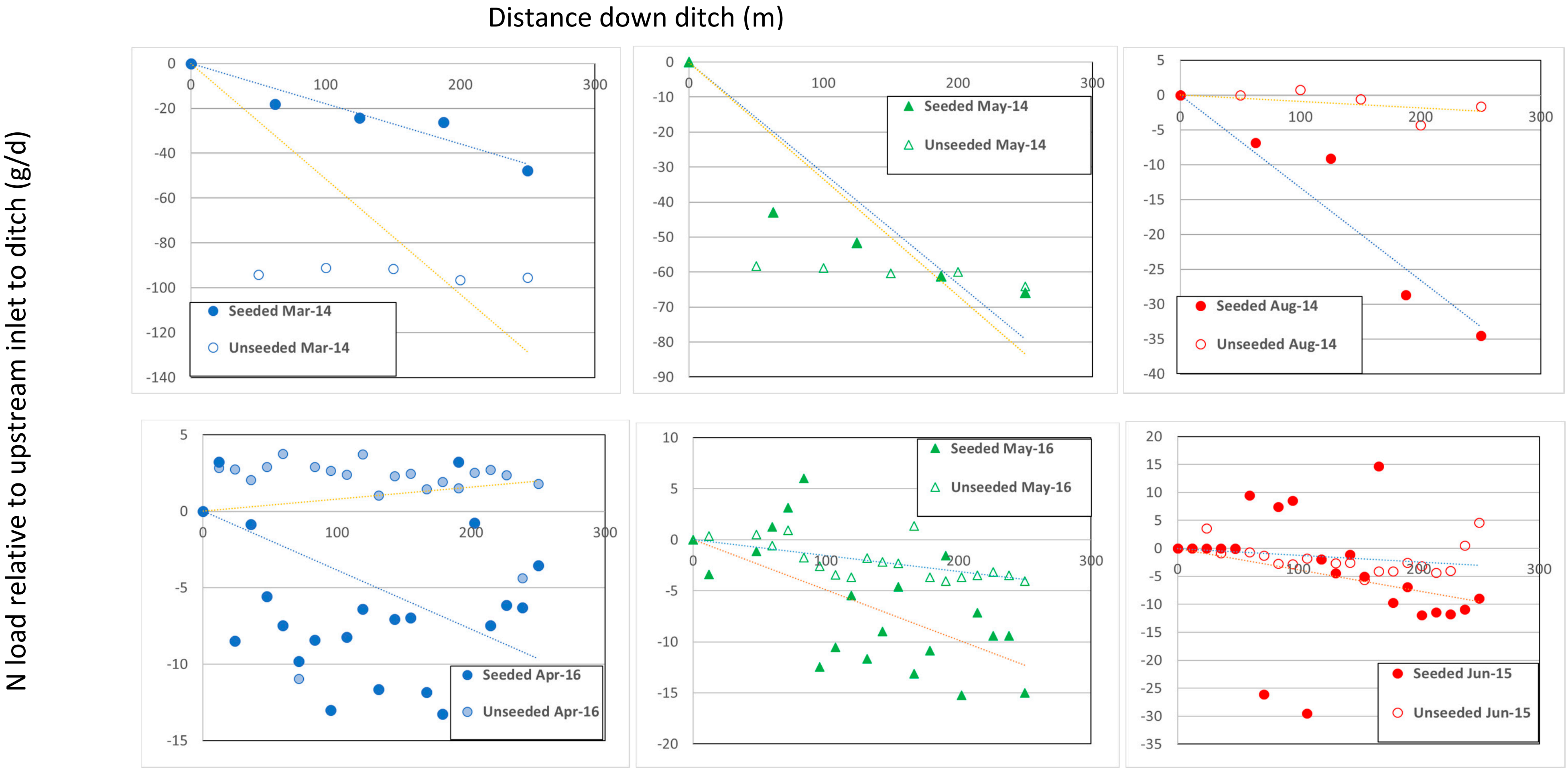

Supplementary data 1b. NO_3-N load change along watercress seeded and unseeded ditches from top end of ditch.

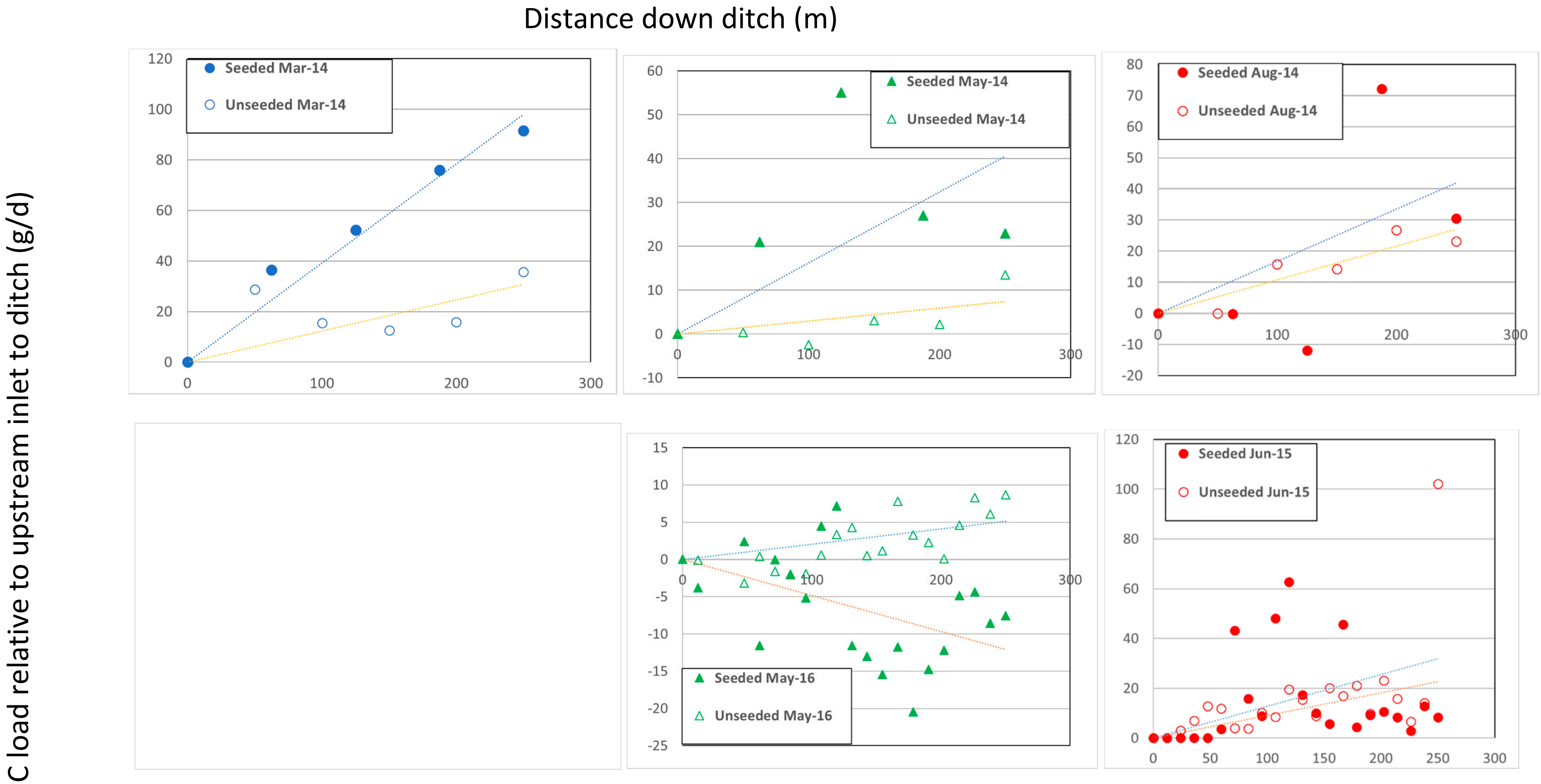

Supplementary data 1c. TOC load change along watercress seeded and unseeded ditches from top end of ditch.

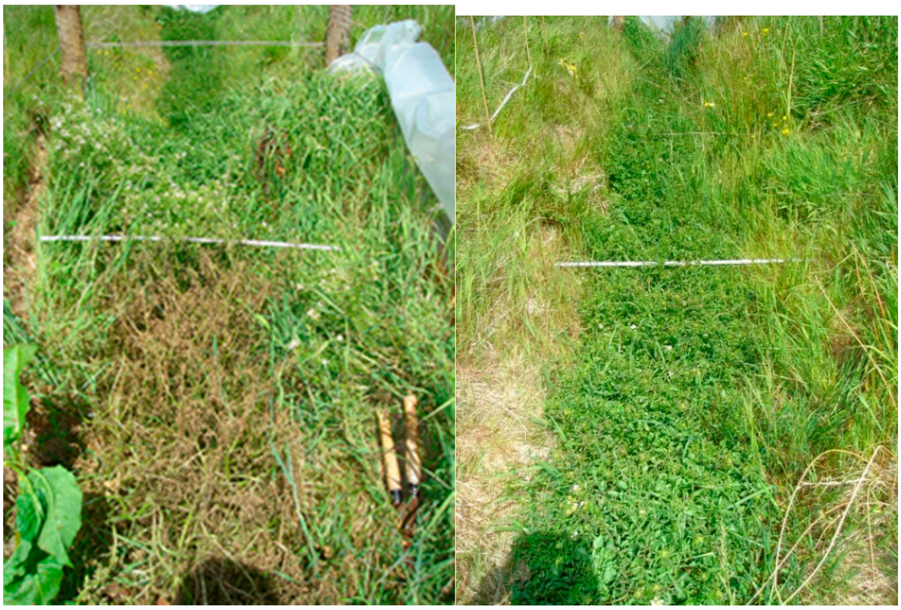

Supplementary information 2a. Seeded covered and seeded uncovered sections, 10 June 2015

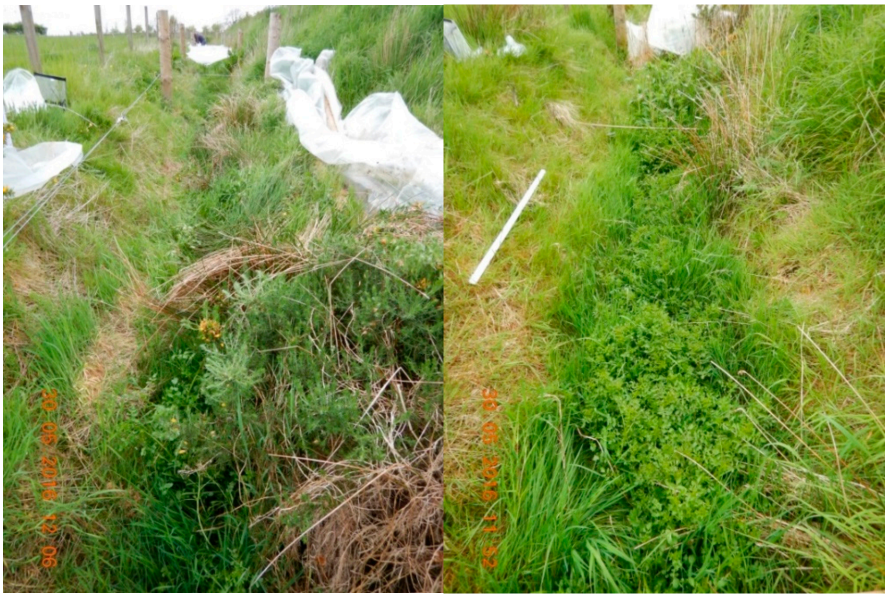

Supplementary information 2b. Section 2 seeded covered and seeded uncovered sections, 30 may 2016

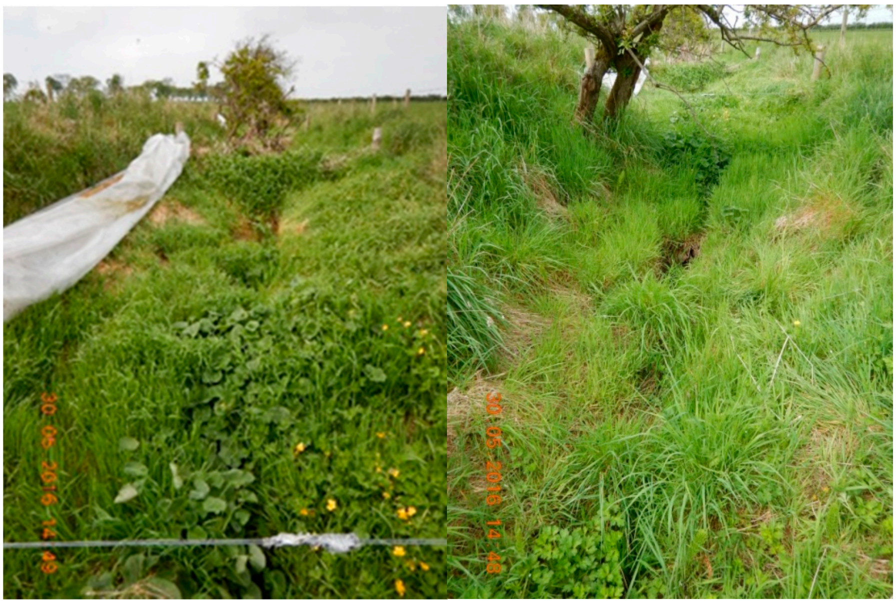

Supplementary information 2c. Section 2 unseeded covered and unseeded uncovered sections, 30 may 2016
